# Supplementary figures and images for: Fgf signalling is required for gill slit formation in the skate, Leucoraja erinacea
Source: Dev Biol. Author manuscript; Available in PMC 2024 Jun 24. (PMC11195640; doi:10.1016/j.ydbio.2023.11.008)

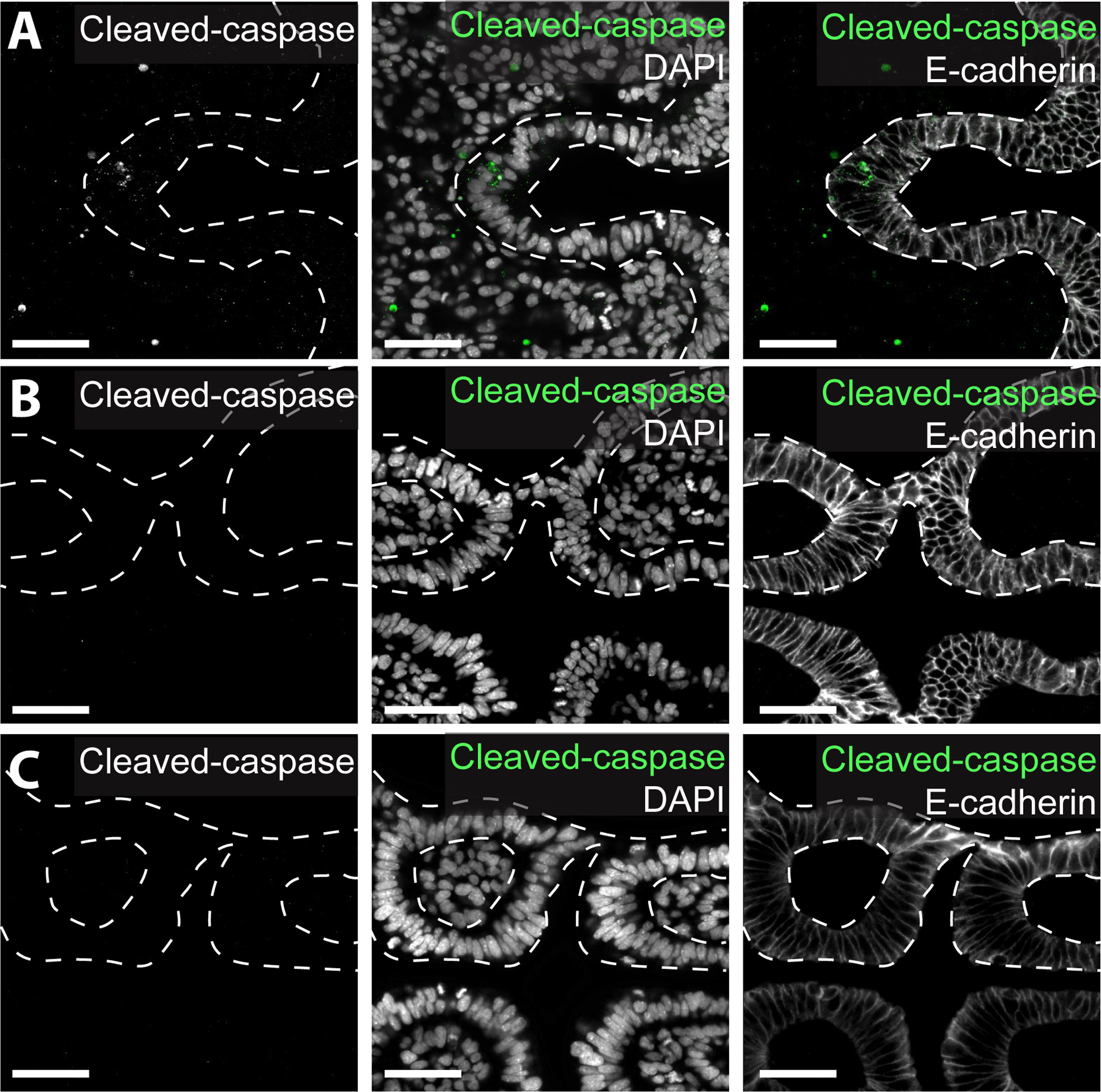

Supplement: Supplemental Fig. 1. No cleaved caspase-3 expression during gill slit formation in the skate. Immunofluorescence for cleaved caspase-3 shows (A) a domain of positive staining in the rostral pharyngeal epithelium (consistent with our observation of TUNEL staining in this region) but (B) no cleaved ca [file NIHMS2000062-supplement-Supplemental_Fig__1__No_cleaved_caspase-3_expression_during_gill_slit_formation_in_the_skate__Immunofluorescence_for_cleaved_caspase-3_shows__A__a_domain_of_positive_staining_in_the_rostral_pharyngeal_epithelium__consi.jpg]
